# Cytisine attenuates bone loss of ovariectomy mouse by preventing RANKL‐induced osteoclastogenesis

**DOI:** 10.1111/jcmm.15622

**Published:** 2020-08-13

**Authors:** Zhi Qian, Zeyuan Zhong, Shuo Ni, Dejian Li, Fangxue Zhang, Ying Zhou, Zhanrong Kang, Jun Qian, Baoqing Yu

**Affiliations:** ^1^ Department of Orthopaedic Surgery Shanghai Pudong Hospital Fudan University Pudong Medical Center Huinan Town China; ^2^ Department of Orthopaedic Surgery Zhangye People's Hospital affiliated to Hexi University Zhangye City China

**Keywords:** AKT‐NFATc1, cytisine, MAPK, NF‐κB, osteoclastogenesis, SC79

## Abstract

Postmenopausal Osteoporosis (PMOP) is oestrogen withdrawal characterized of much production and activation by osteoclast in the elderly female. Cytisine is a quinolizidine alkaloid that comes from seeds or other plants of the Leguminosae (Fabaceae) family. Cytisine has been shown several potential pharmacological functions. However, its effects on PMOP remain unknown. This study designed to explore whether Cytisine is able to suppress RANKL‐induced osteoclastogenesis and prevent the bone loss induced by oestrogen deficiency in ovariectomized (OVX) mice. In this study, we investigated the effect of Cytisine on RAW 264.7 cells and bone marrow monocytes (BMMs) derived osteoclast culture system in vitro and observed the effect of Cytisine on ovariectomized (OVX) mice model to imitate postmenopausal osteoporosis in vivo. We found that Cytisine inhibited F‐actin ring formation and tartrate‐resistant acid phosphatase (TRAP) staining in dose‐dependent ways, as well as bone resorption by pit formation assays. For molecular mechanism, Cytisine suppressed RANK‐related trigger RANKL by phosphorylation JNK/ERK/p38‐MAPK, IκBα/p65‐NF‐κB, and PI3K/AKT axis and significantly inhibited these signalling pathways. However, the suppression of PI3K‐AKT‐NFATc1 axis was rescued by AKT activator SC79. Meanwhile, Cytisine inhibited RANKL‐induced RANK‐TRAF6 association and RANKL‐related gene and protein markers such as NFATc1, Cathepsin K, MMP‐9 and TRAP. Our study indicated that Cytisine could suppress bone loss in OVX mouse through inhibited osteoclastogenesis. All data provide the evidence that Cytisine may be a promising agent in the treatment of osteoclast‐related diseases such as osteoporosis.

## 1 INTRODUCTION

Bone maintains in a homeostasis because of osteogenesis of osteoblast and osteoclastogenesis of osteoclast.^1^ Once this condition upset and there will occur bone diseases, such as osteoporosis, osteopetrosis, rheumatoid arthritis and so on. Osteoporosis is a metabolic disease that could diminish bone mineral density, alter micro‐structure and lead to much bone loss. Postmenopausal osteoporosis (PMOP) is the most familiar disease that occurred in older women owing to an imbalanced situation between osteoblast and osteoclast. PMOP usually results in osteoporotic fracture and body pain.^2^ Oestrogen is very important for osteoprotectiving that can urge osteoblasts to secrete osteoprotegerin (OPG) and attenuate osteoclastogenesis, osteoclast precursor differentiation and osteoporosis formation.^3^ Oestrogen deficiency has become an independent key factor of pathologic PMOP condition.^4^ So, it is a wise therapeutic way to restrain osteoclast differentiation and formation by diminishing the progress of PMOP.^5^

Osteoclasts are multinucleated huge cells that result from a monocyte‐macrophage lineage by stimulation of macrophage colony‐stimulating factor (M‐CSF) and receptor activator of nuclear factor‐κB ligand (RANKL).^6^ The cytokine M‐CSF is a basis of osteoclast precursor to proliferation and survival; meanwhile, cytokine RANKL which comes from a tumour necrosis factor (TNF) family that regulates the interaction with its receptor RANK to exert the function of becoming mature osteoclast.^7^ With the binding of M‐CSF and extracellular signal‐related kinase (ERK 1/2), osteoclast precursor gradual proliferation and survival.^8^ With the binding of RANKL and its receptor RANK that recruit tumour necrosis factor receptor‐associated factors (TRAFs), many intracellular signalling pathways were activated, including PI3K‐AKT and ERK 1/2‐MAPK signalling.^9^ Nuclear factor of activated T‐cell cytoplasmic 1 (NFATc1) is the vital transcriptional factor for osteoclast and its related transcriptional genes, such as matrix metalloproteinase 9 (MMP‐9), cathepsin K (CTSK), calcitonin receptor (CTR) and tartrate‐resistant acid phosphatase (TRAP).^10^ Subsequently, the NFATc1 and osteoclast are activated and promoted intracellular Ca^2+^ releasing.^11^ Thus, based on former works, suppressing RANKL‐induced osteoclastogenesis maybe a good idea to cure PMOP.

Cytisine is a quinolizidine alkaloid that comes from seeds or other plants of the Leguminosae (Fabaceae).^12^ It has been used to as a partial agonist of high‐affinity acetylcholine receptors to withdraw nicotine cessation.^13^ From the former research, we found that Cytisine has several pharmacological functions, such as anti‐depressant like property,^14^ anti‐diabetes,^15^ antivirus,^16^ against human influenza virus A (H1N1)^16^ and against cerebral ischemia‐reperfusion^17^ properties and so on. Furthermore, there has been reported that Cytisine alleviated liver fibrosis via PI3K/AKT/Smad pathway.^18^ In addition, Cytisine has been implied to exert anti‐tumour effects on lung cancer cells by regulating reactive oxygen species‐induced and MAPK, NF‐κB signalling pathways^19^ and inhibited human breast cancer cells through apoptosis‐induced activity.^20^ However, both the function of treatment for osteoporosis and the mechanism by Cytisine suppresses OCs have not yet been studied. So, we managed this study to detect whether Cytisine has the protecting function of ovariectomy‐induced bone loss and the possible deeper molecular mechanism.

In this study, we illustrated the phenomenon of Cytisine suppressed RANKL‐induced osteoclastogenesis and its possible mechanism in vitro. We implied that Cytisine inhibited osteoclast‐precursor become mature osteoclast and decreased bone absorption via impacting NF‐ κB, MAPKs and PI3K/AKT signalling pathways. In addition, our work showed that Cytisine can take a positive role in decreasing bone loss at oestrogen deficiency‐induced osteoporosis mouse model. Thereby, our research showed that Cytisine may have latent effects against osteolytic diseases, implying its value of curing for osteoporosis.

## 2 MATERIALS AND METHODS

### 2.1 Ethics statement

Our study was approved by the Animal Care and Use Committee at the Fudan University Hospital Medical Center. All of the surgical operation, therapy and post‐operative animal care steps were executed to keep up with the National Institutes of Health (NIH) Guide for the Care and Use of Laboratory Animals.

### 2.2 Reagents and antibodies

Cytisine (pure > 98%) which was purchased from NatureStandard (Shanghai Standard Technology Co.,Ltd, China) and was dissolved in DMSO at a 100 mmol/L stock solution and stored at refrigerator (−20℃). Further dilution was carried out in a culture medium for cells and plant oil medium for animals. Primary antibodies against Cathepsin K, NFATc1, CTR, MMP‐9, TRAP and β‐actin were purchased from Proteintech Group (Proteintech Group, Inc., Rosemont, IL, USA). Primary antibodies against JNK, p‐JNK, ERK, p‐ERK, p38, p‐p38, IκBα, p‐ IκBα, p65, p‐p65, PI3K, p‐PI3K, AKT, p‐AKT, NFATc1 and GAPDH were obtained from Cell Signaling Technologies (Beverly, MA, USA). The activator SC79 of AKT was purchased from MCE (MedChemExpress Co, Monmouth Junction, NJ, USA). Anti‐RANK and anti‐TRAF6 were purchased from Abcam (Cambridge, UK), Protein A/G agarose beads were come from Santa Cruz Biotechnology (Santa Cruz, CA, USA). EP‐40 assay buffer was purchased at Solarbio Life Sciences (Beijing Solarbio Science & Technology Co., Ltd. China). A CCK‐8 assay kit was purchased from DoJinDo (DoJinDo ChemTech Lim, Japanese). A tartrate acid phosphatase staining kit was obtained from Sigma‐Aldrich (Sydney, Australia). Recombinant M‐CSF and m‐RANKL were obtained from R&D Systems (Minneapolis, MN, USA). The cell culture medium that alpha‐modified minimal essential medium (α‐MEM), foetal bovine serum (FBS) and penicillin‐streptomycin were purchased from Thermo Fisher Scientific (Carlsbad, CO, USA).

### 2.3 Cell viability assay

We used a cell counting kit‐8 (CCK‐8) assay to detect cell viability at the guide of manufacturing protocol. The cells were cultured into 96‐well plates (5 × 10^3^ cells/well for RAW 264.7 and 1 × 10^4^ cells/well for BMMs, respectively) and incubated with RANKL (50 ng/mL) and different concentration of Cytisine at 37℃ for 24, 48 and 96 hours. After every time point treatment that the plate was washed with PBS and added 100 μL/well CCK‐8 reagent and incubated at 37℃ for 1 hour. The absorbance of the wells was detected at 490 nm by a micro‐plate reader (Thermo Fisher, Waltham, MA, USA).

### 2.4 In vitro osteogenesis assay

To detect the function of Cytisine on the differentiation and mineralization of osteoblast, BMSCs cells were isolated from femurs of six‐week‐old C57BL/6 mice. Bone marrow was washed by PBS till the bone marrow cavity bleached and sent to centrifugate (1000 g, 10 minutes, 4℃). The extracted cells were planted at 9 cm dish plates for 24 hours, then collected the unattached cells for osteoclastogenesis assay and the adherent cells passaged 2‐3 generations for the present study. The BMSCs' differentiation to osteoblast was induced by complex culture medium (α‐MEM media with 10% FBS, 1% antibiotic mixture of penicillin and streptomycin, 100 nmol/L dexamethasone, 50 µmol/L ascorbic acid and 10 mmol/L glycerophosphate). Cytisine (25 μmol/L and 12.5 μmol/L) was added into the above medium during the differentiation. Alkaline phosphatase (ALP) assay and alizarin staining assay were executed to mark the osteoblast differentiation and mineralization through ALP and alizarin staining kit (Beyotime Biotechnology, Shanghai, China) separately.

### 2.5 In vitro osteoclastogenesis assay

RAW264.7 cells were obtained from Ph.D Li (East China Normal University). Bone marrow macrophages (BMMs) cells were isolated from the femoral bone marrow of C57BL/6 mice at six‐week‐old by centrifugation (10 000 g, 45 seconds, 4℃). The extracted cells were lysed with red blood cell lysate, were isolated by centrifugation again (400 g, 8 minutes, 21℃) and cultured in a cell dish (9 cm, diameter) with α‐MEM basic medium (contained 1 × glutamine, 5 ng/mL M‐CSF and 1% antibiotic mixture of penicillin and streptomycin) in a cell culture incubator (5% CO_2_, 37℃) for 24 hours. Then collected the non‐adhesive cells and planted at a density of 5 × 10^4^ cells/well onto 48‐wells plates with α‐MEM basic medium for three days. Then, the cell culture medium was altered every two days by α‐MEM complete medium (contained 90% α‐MEM, 10% FBS and 1% antibiotic mixture of penicillin and streptomycin) and M‐CSF (10 ng/mL) till the cells proliferated at 80%–90% confluence. RAW264.7 cells were planted at a density of 1 × 10^4^ cells/well in 48‐wells plates depend on α‐MEM complete culture medium (adding 90% α‐MEM, 10% foetal bovine serum and 1% antibiotic mixture of penicillin and streptomycin) including M‐CSF (10 ng/mL) and RANKL (50 ng/mL). The third passage BMMs and RAW264.7 cells were divided into control group and four treatment groups with Cytisine (0, 6.25,12.5, 25 μmol/L). The osteoclasts began to occur at four and seven days of RAW264.7 and BMMs cells, respectively. Then, the osteoclasts were stained by tartrate‐resistant acid phosphatase (TRAP) staining kit (Sigma‐Aldrich, St. Louis, MO, USA) in accordance with protocol of manufacture. TRAP‐positive multinucleated cells (>3 for BMMs, >5 for RAW264.7 cells) were regarded as osteoclast cells, and the number was counted at microscope (Nikon Corporation, Tokyo, Japan).

### 2.6 F‐actin ring formation assay

BMMs cells were planted at 48‐well plates and treated with various concentrations of Cytisine and contained with M‐CSF and RANKL (10 ng/mL and 50 ng/mL, respectively). About 5 days later, the BMMs cells translated into osteoclast which fixed by paraformaldehyde (4%) for 10 minutes at room temperature. Then, wells of plates were washed with PBS for three times, the cells were permeated by 0.1% triton‐X 100 for 5 minutes and blocked with 3% BSA. Then, the cells incubated with rhodamine‐conjugated phalloidin (Solarbio, Co., Ltd., Beijing, China) and DAPI (Solarbio, Co., Ltd. Beijing, China) to detect F‐actin ring and the cells nuclei, respectively, at confocal laser scanning microscope (Nikon Corporation, Tokyo, Japan). The number of nuclei and F‐actin ring was counted.

### 2.7 Pit‐resorption assay

A pit‐resorption assay was executed to assess the function of osteoclast cells. BMMs cells were planted into bone biomimetic synthetic surface (Osteo Assay Surface 24‐well plates, Corning, NY, USA) and bone slices (Immunodiagnostic Systems Limited, IDS Ltd, Boldon, UK) on density of 1 × 10^5^ cells/mL and incubated with M‐CSF (10 ng/mL) and RANKL (50 ng/mL) till osteoclast precursor occurred. Then the pre‐osteoclast cells were treated with various concentrations of Cytisine as record above. After 2 days, most of the bone biomimetic synthetic surface were bleached, to remove the bone slices and dried, while the remain wells of 24‐well plates were fixed and stained by TRAP staining as stated before to estimate the number of osteoclast cells. Besides, the bone slices were stained by toluidine blue O (Solarbio, Co., Ltd. Beijing, China) staining to survey the area of pit resorption which was calculated by Image J software (NIH, Bethesda, MD, USA).

### 2.8 Real‐time PCR

Quantitative real‐time polymerase chain reaction (qRT‐PCR) was performed to determine mRNA expression levels of the RNAKL‐induced osteoclast specific genes NFATc1, Cathepsin K, MMP9 and TRAcP. The RAW264.7 cells disposed with or without various concentrations of Cytisine (12.5, 25 μmol/L) in presence of M‐CSF (10 ng/mL) and RANKL (50 ng/mL) in 6‐well plates and the total RAN of cells were extracted through Trizol reagent (life, Life Technologies, AB & Invitrogen, Carlsbad, USA). Later, 1 μg of total RNA was reversed to synthesize cDNA through a RT‐PCR kit (Invitrogen, Carlsbad, USA). Procedures of PT‐PCR were 5 minutes at 94℃, followed by 30 cycles of 40 seconds at 94℃, 40 seconds at 60℃ and 40 seconds at 72℃, and the final extension movement of 5 minutes at 72℃. The reaction was managed through a ViiA^TM^ 7 Real‐time PCR equipment (Applied Biosystem, Paisley, UK). Last, to collect and normalize the cycle threshold to the level of GAPDH. A data were analysed by 2^‐△△CT^ method. The specific used primers were listed in Table 1.

**TABLE 1:** Primer sequence used in qRT‐PCR

Gene	Forward primer	Reverse primer
Cathepsin K	TAGCACCCTTAGTCTTCCGC	CTTGAACACCCACATCCTGC
MMP‐9	CGACTTTTGTGGTCTTCCCC	TAGCGGTACAAGTATGCCTCTG
TRAP	TGGGTGACCTGGGATGGATT	AGCCACAAATCTCAGGGTGG
NFATc1	CCAGCTTTCCAGTCCCTTCC	ACTGTAGTGTTCTTCCTCGGC
GAPDH	AGGAGAGTGTTTCCTCGTCC	TGAGGTCAATGAAGGGGTCG

### 2.9 Western blotting

To determine the effect of Cytisine on RANKL‐induced osteoclast specific proteins (NFATc1, Cathepsin K, CTR, MMP9 and TRAP), JNK/ERK/p38‐MAPK, IκBα/p65‐NF‐κB and PI3K‐AKT pathway in RAW264.7 cells through Western blotting. The RAW264.7 cells were planted into 6‐well plates (5 × 10^4^ cells/well) and divided into two groups: the former group cells were treated with RANKL and determined by Western blotting at different time points (0, 1, 3, 5 days) to survey the RANKL‐induced osteoclast protein levels as mentioned above, the other group cells were treated with RANKL and two concentrations of Cytisine (0, 25 μmol/L) and performed by Western blotting at different time points (0, 15, 30, 60 minutes) to observe phosphorylation of JNK/ERK/p38‐MAPK, IκBα/p65‐NF‐κB, PI3K/AKT and total NFATc1. The treated RAW264.7 cells were isolated by radioimmunoprecipitation assay (RIPA) lysis buffer (Teye, Bioteke Cor. Beijing, China) on ice. Equal amount of osteoclast‐related protein was separated by sodium dodecyl sulphate‐polyacrylamide gel electrophoresis (SDS‐PAGE) and transferred to polyvinylidene fluoride (PVDF) membranes (GE Healthcare, Silverwater, Australia). The membranes were blocked with 5% skim milk at room temperature for 2 hours and incubated at 4℃ overnight with one of the following two groups primary antibodies: NFATc1, CTR, MMP9, TRAP and CTSK for first group while p‐JNK, p‐ERK, p‐p38‐MAPK, p‐IκBα, p‐p65(NF‐κB), p‐PI3K, p‐AKT and NFATc1 for second group. Subsequently, membranes were incubated with accordance of secondary antibodies for 1 hour at room temperature. Protein bands were tested through an enhanced chemiluminescence (ECL) liquid (Teye, Bioteke Cor. Beijing, China) and system (Amersham Pharmacia Biotech, Sydney, NSW, Australia).

### 2.10 Immunoprecipitation

To make sure whether Cytisine can impact RANKL‐induced relationship between RANK and TRAF6, the immunoprecipitation was performed. In a word, RAW264.7 cells (1 × 10^6^ cells/well) were planted into a 6 cm‐dish and cultured with or without Cytisine 25 μmol/L for 3 hours. After stimulated by RANKL (100 ng/mL) for a half hour, then all the cells were lysed in EP‐40 assay buffer and centrifuged at 6000 *g* for 10 minutes at 4℃. Take out of 50 μL supernatant liquid protein of per micro‐tube for WB Input assay, then the RANK or TRAF6 antibody was added into the remaindered supernatant and incubated at 4℃ overnight with rotation. Protein A or G agarose beads were added and cultured at 4℃ for 3 hours with rotation. After centrifugation (1100 *g*, 4℃, 5 minutes) at 4 times, added loading buffer and boiled protein at 95℃ which the proteins were collected to Western blot.

### 2.11 Animals and experiments

All experiments were executed in the Specific Pathogen Free (SPF) laboratory of Fudan University Pudong Medical Center. Female six‐week‐old C57BL/6 mice were obtained from Shanghai Lab. Animal Research Center (Shanghai, China) and kept under standard conditions with free access to clean water and food. All animals were randomly divided into three groups (sham group, OVX group, and OVX + Cytisine group, n = 6) and anaesthetized intraperitoneally with 2% (w/v) pentobarbital (40 mg/kg). The bilateral dorsal approach of OVX step was performed between OVX group and OVX + Cytisine group which two ovaries and part of the oviduct were excised and pressed to stop any bleeding. The sham group mice were performed the same incision and only removed little fatty tissue around the ovaries. To suture the skin incision via 5‐0 non‐absorbent silk threads and mice were allowed to recover for three days at post‐operation. The OVX + Cytisine group mice were received Cytisine (25 mg/kg) dissolved in DMSO (C_DMSO_ < 0.2%) and plant oil through intraperitoneal administration every other day for eight successive weeks. The sham group and OVX group mice were executed an equivalent volume medium of PBS. After 8 weeks treatments, all mice were anaesthetized with pentobarbital (2% w/v); bilateral femur bone and arterial blood were collected for posterior experiments.

### 2.12 Bone histological analysis

The left femurs were excised from all mice and fixed in 4% paraformaldehyde at room temperature for 4 days and decalcified in 10% tetrasodium‐ethylenediaminetetraacetic acid (T‐EDTA) for 2 weeks. Then, femur bone samples were paraffin‐embedded, sectioned 4 μm thickness and prepared with a microtome (Jung, Heidelberg, Germany). Haematoxylin and eosin (H&E) were executed to measure trabecular bone. TRAP staining was performed to detect osteoclast in the distal femur metaphysis bone tissue. All standard histologic bone slices measures were analysed by microscope which primary magnification was × 10 (BX53, Olympus), and all data were calculated by Image J software (NIH).

### 2.13 Miro‐computed tomography analysis

The right femurs were measured by micro‐computed tomography (Skyscan1172, Bruker, Kontich, Belgium). The trabecular bone selected from distal femur metaphysis were scanned at following parameters: 50 kV for tube voltage, 500 μA for current, 9 μm for voxel size in reconstructed image. Images were analysed by a plug‐in program within the following histological parameters for trabecular bone of distal femur metaphysis: bone volume/ total volume (BV/TV), bone surface area/total volume (BS/TV), bone surface/ volume ratio (BS/BV), bone mineral density (BMD), trabecular number (Tb. N) and Trabecular pattern factor (Tb.Pf). Last, the two‐dimensional (2D) and three‐dimensional (3D) structure of the distal femur were built‐in software.

### 2.14 Statistical analysis

All experiments were performed at least three times. All results were expressed as means ± SD. Statistical analysis was measured by GraphPad Prism version 7.0 software (GraphPad software, San Diego, CA, USA). Intergroup and intragroup comparisons were executed using one‐way ANOVA followed by Tukey test. Values of *P* < 0.05 were considered statistically significant.

## 3 RESULTS

### 3.1 Cytisine inhibited BMMs and RAW264.7 cells into osteoclast in vitro

**FIGURE 1:**
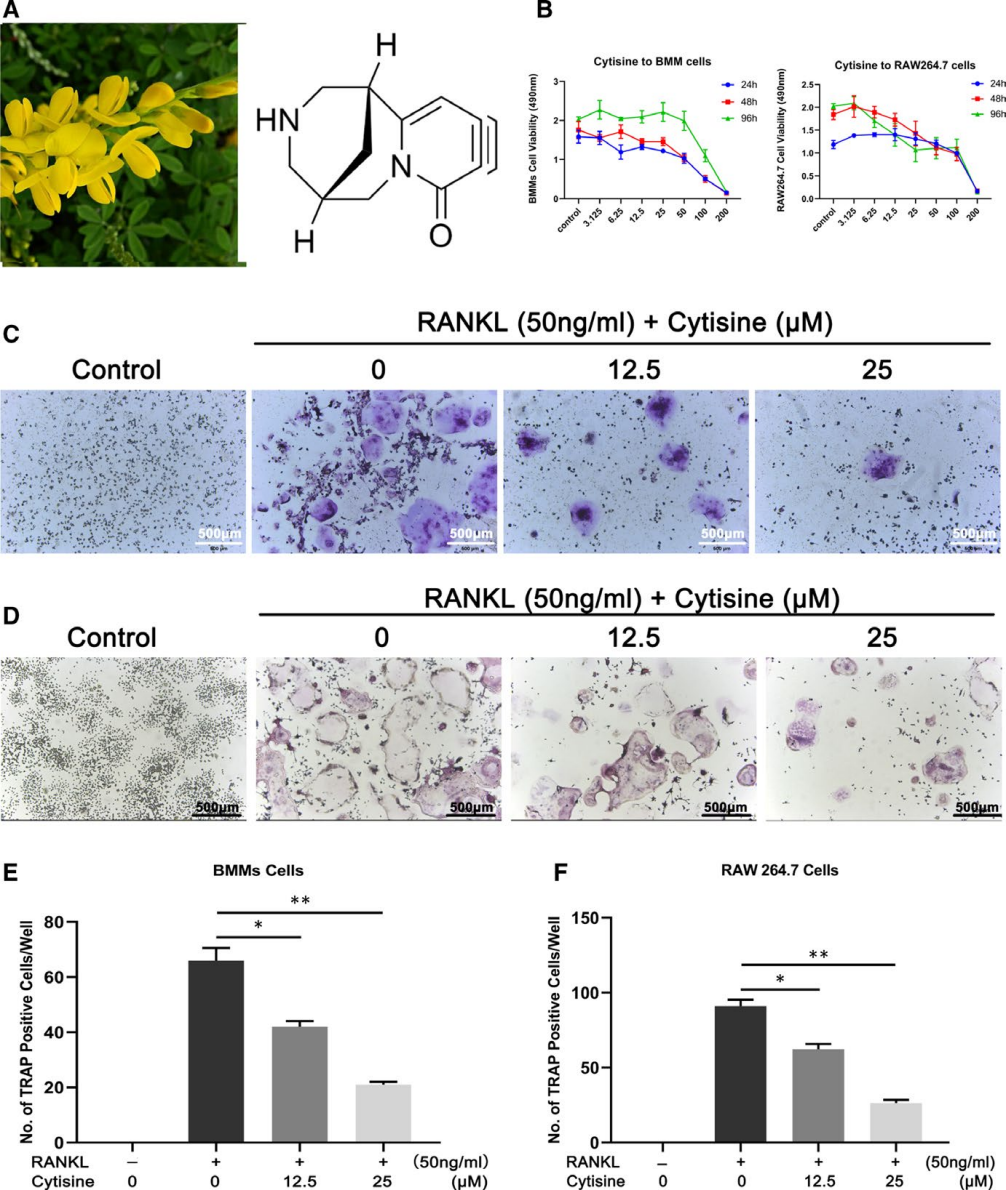
Cytisine inhibited BMMs and RAW264.7 cells into osteoclast in vitro. A, The chemical structure of Cytisine; B, The cytotoxicity of various concentrations of Cytisine on BMMs and RAW264.7 cells in different time was performed by CCK‐8 assay; C, Representative of TRAP‐positive images of osteoclast from BMMs cells onto different concentrations of Cytisine. Scale bar were 500 μm; D, Representative of TRAP‐positive images of osteoclast from RAW264.7 cells onto different concentrations of Cytisine. Scale bar were 500 μm; (E,F) The quantification of TRAP‐positive multinuclear cells (nuclei > 3 of BMMs and nuclei > 5 of RAW264.7 cells) were shown in C and D. Data are presented with mean ± SD, *P < 0.05; **P < 0.01

The chemical structure of Cytisine is shown in Figure 1A. To examine the cytotoxic effect of Cytisine contributed to suppressing osteoclastogenesis, the cytotoxicity of Cytisine (0, 3.125, 6.25, 12.5, 25, 50, 100, 200 μmol/L) on BMMs and RAW264.7 cells were performed after 24, 48, 96 hours treatment by CCK‐8 assay kit (Figure 1B). The Cytisine has obvious cytotoxic to BMMs and RAW264.7 cells when concentrations up to 200 μmol/L indicated that no significant cytotoxicity was surveyed of Cytisine less than 200 μmol/L for BMMs and RAW264.7 cells at the concentration above. Meanwhile, we found that among the Cytisine dose range of 0‐25 µmol/L, relative to the control group that Cytisine did not decrease the number of BMMs and RAW264.7 cells. So, we selected this dose range for our study.

To explore the effect of Cytisine to RANKL‐induced osteoclastogenesis, we used two standard osteoclast differentiation in vitro. RAW264.7 cells were treated with RANKL while BMMs cells were treated with M‐CSF and RANKL in the different concentration presence of Cytisine. The total number of TRAP‐positive multinucleated cells (>3 nuclei for BMMs, >5 nuclei for RAW264.7 cells) was significantly decreased as Cytisine concentration increased from 0 to 25 μmol/L compared with the control group (*P* < 0.05), especially, the 12.5 μmol/L group were significantly more than 25 μmol/L group (*P* < 0.05). The results implied that osteoclast differentiation and formation of both BMMs cells (Figure 1C,E) and RAW264.7 cells (Figure 1D,F) were diminished by Cytisine in a concentration‐dependent manner from 0 to 25 μmol/L.

### 3.2 Cytisine suppressed RANKL‐induced osteoclast differentiation only at early stage

**FIGURE 2:**
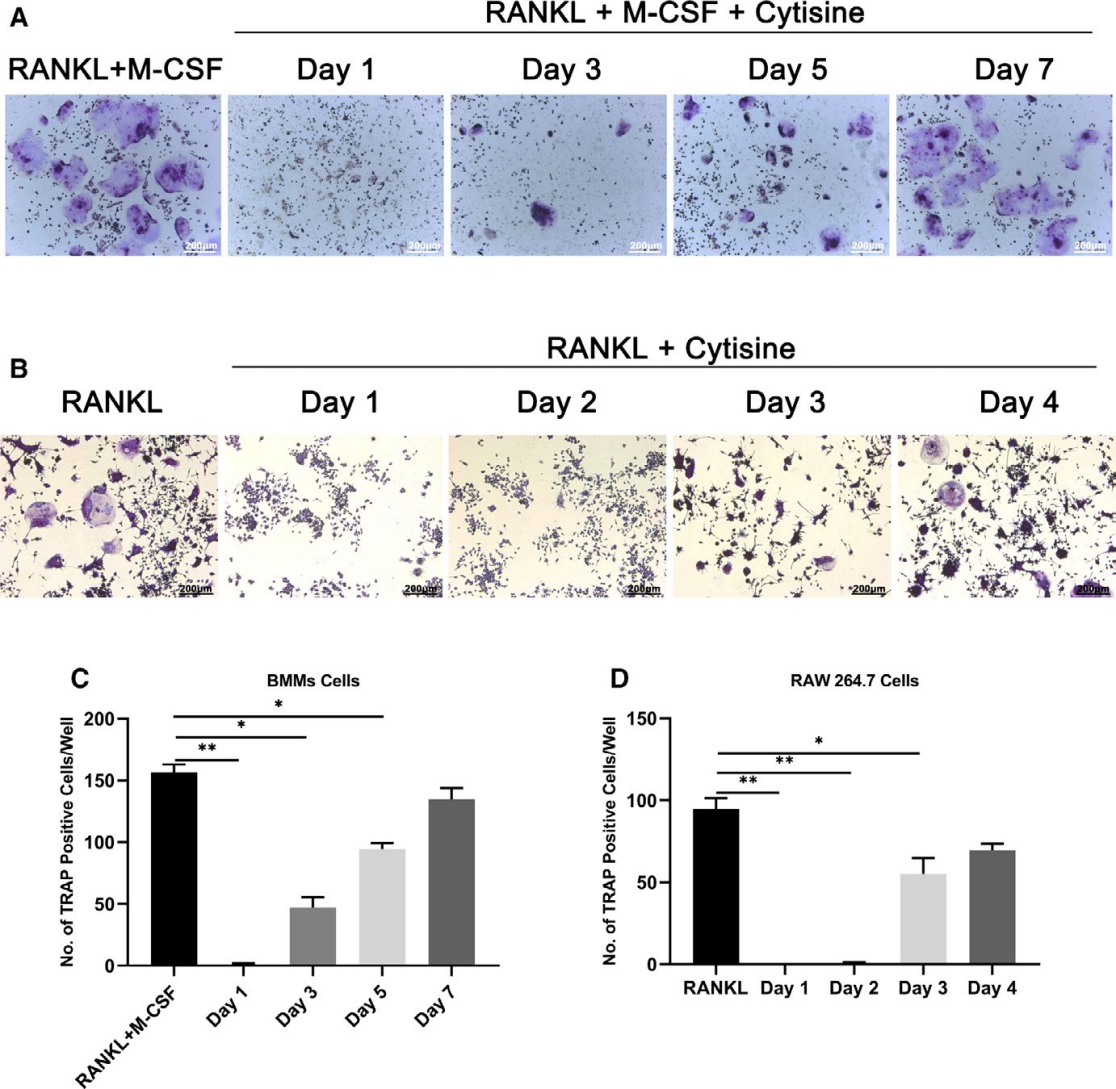
Cytisine suppressed RANKL‐induced osteoclast differentiation only at early stage. A, Cytisine planted into culture medium at different time point for BMMs cells. B, Cytisine seeded into culture medium at different time point for RAW264.7 cells. (C & D) The quantification of TRAP‐positive multinuclear cells (nuclei > 3 of BMMs and nuclei > 5 of RAW264.7 cells) were shown in A and B. Data are presented with mean ± SD, *P < 0.05; **P < 0.01

Bone marrow monocytes cells or RAW264.7 cells transfer osteoclast is a multistep progress which contains proliferation, differentiation, cell fusion and multinucleation and so on. It is necessary to detect which stage that Cytisine could suppress osteoclast differentiation. The Cytisine was added to culture at beginning on day 1 to day 7 for BMMs cells (Figure 2A,C) and day 1 to day 4 for RAW264.7 cells (Figure 2B,D). The results showed that Cytisine inhibited osteoclastogenesis totally at day 1 to day 3 for BMMs cells and day 1 to day 2 for RAW264.7 cells; meanwhile, there was no suppressible effect to exposure precursor osteoclast at later stage.

### 3.3 Cytisine suppressed RANKL‐induced F‐actin ring and bone‐pit formation

**FIGURE 3:**
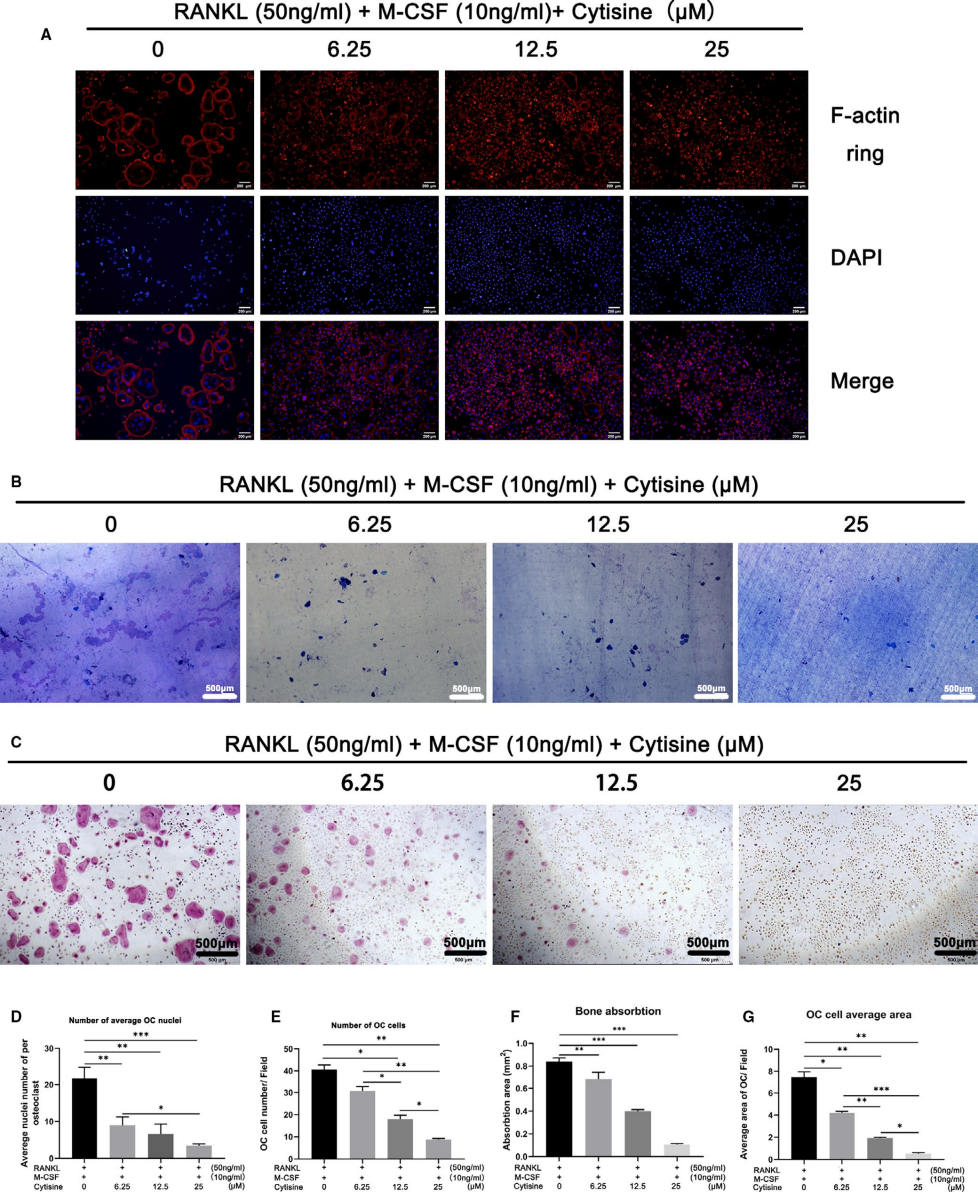
Cytisine suppressed RANKL‐induced F‐actin ring and bone‐pit formation. A, The F‐actin ring formation was performed in various concentration of Cytisine by immunofluorescence combined with DAPI staining in nuclei. Scale bar were 200 μm; B, Representative images of bone‐pit resorption in bone slices. Scale bar were 200 μm; C, Representative images of TRAP‐positive osteoclast in hydroxyapatite‐coated plates. Scale bar were 500 μm; (D,E) Quantification of osteoclast per field and average nuclei number of each osteoclast treated with various concentration of Cytisine. (F,G) Quantification of bone‐pit resorption area and osteoclast number per well under the different concentration of Cytisine. Data are presented with mean ± SD, *P < 0.05; **P < 0.01; ***P < 0.001

To further examine the effect of Cytisine on osteoclastogenesis, we determined whether Cytisine effected osteoclast fibrous actin (F‐actin) ring formation. The F‐actin ring is a cytoskeletal structure which is the most obvious characteristic of osteoclast for bone resorption. BMMs cells differentiated into mature osteoclast when incubated with M‐CSF and RANKL by staining with rhodamine‐conjugated phalloidin and DAPI (Figure 3A,D,E). Whereas, the size and number of F‐actin ring structures were significantly diminished when the cells were treated with Cytisine, and we could conclude that Cytisine inhibited the formation of F‐actin rings in mature osteoclast.

To deep examine, whether Cytisine suppressed bone pit formation function by using biomimetic synthetic bone surface of osteoclast. BMMs cells were planted into 48‐well plates which coated with bone slices and treated with or without Cytisine for 72 hours. The osteoclast activity was weakened obviously owning to Cytisine treatment, and the pit‐forming of bone slices was inhibited severely at 12.5 μmol/L and 25 μmol/L (Figure 3B,F,G). The same situation of TRAP staining for OCs of bone slices (Figure 3C). Therefore, the bone pit formation activity and TRAP staining for hydroxyapatite‐coated plates of the mature osteoclast were both attenuated by Cytisine.

### 3.4 Cytisine had no effect on differentiation and mineralization of osteogenesis in vitro

As is known, bone sustains homeostasis and remodelling owning to a balance which between osteogenesis of osteoblast and osteoclastogenesis of osteoclast. To explore the effect of differentiation on osteoblast, we executed ALP and alizarin red staining assay for BMSCs cells. As results displayed, there was no significant effect differentiation and mineralization compared with control group of Cytisine on osteoblast (Figure S1A,B) by using Cytisine at experiment dosage range of 0 to 25 μmol/L (Figure S1C,D). Thus, all data demonstrated that Cytisine had no effect of osteogenesis on differentiation and mineralization in vitro.

### 3.5 Cytisine suppressed RANKL‐induced osteoclast‐related gene expression

**FIGURE 4:**
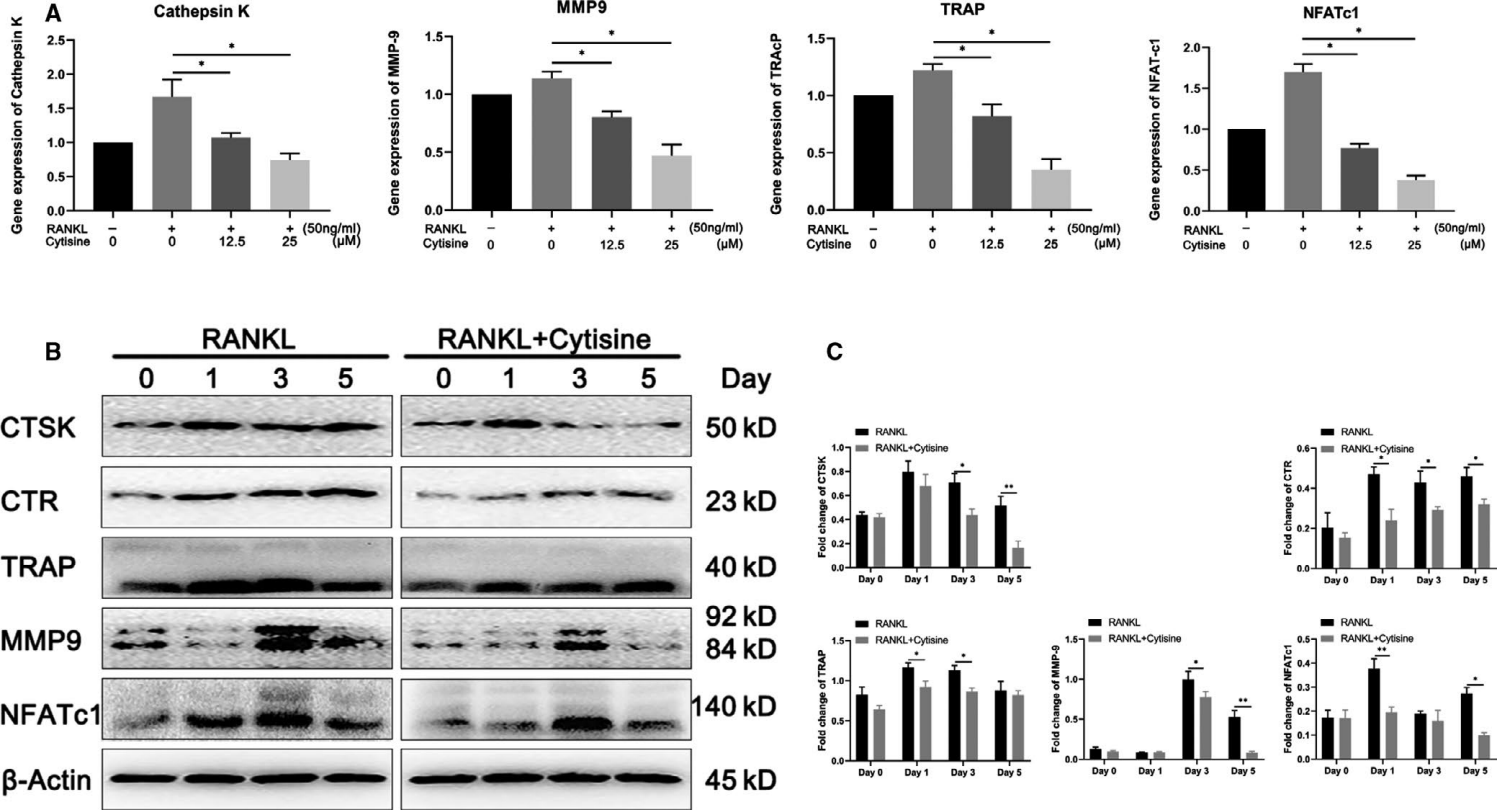
Cytisine down‐regulated osteoclastogenesis gene and protein marker expression. A, Real‐time PCR was performed onto RAW264.7 cells to detect Cytisine with or without RANKL that focused on osteoclast‐related gene expression of Cathepsin K, MMP‐9, TRAP and NFATc1. The expression levels of these genes were normalized with GAPDH. B, Western blotting was measured to discover Cytisine with or without RANKL onto RAW264.7 cells that focused on osteoclast‐related protein marker expression of Cathepsin K, CTR, TRAP, MMP‐9 and NFATc1 at different time point. C, Quantification of osteoclast‐related protein expression at different time point according to Western blotting. Data are presented with mean ± SD, *P < 0.05; **P < 0.01; ***P < 0.001

Osteoclast differentiation and function because of the expression of some related symbol gene, such as NFATc1, Cathepsin K, MMP‐9 and TRAP, which base sequence was listed in Table. 1. We determined the infect of Cytisine on RANKL‐induced osteoclastogenic gene expression by real‐time PCR. RAW264.7 cells were incubated with or without RANKL at various dosage. As is shown, Cytisine significantly attenuated RANKL‐induced up‐regulation of NFATc1, Cathepsin K, MMP‐9 and TRAP gene expression on a concentration‐dependent way (Figure 4A). Thus, Cytisine significant suppressed RANKL‐induced up‐regulation of osteoclastogenesis‐related gene expression.

### 3.6 Cytisine suppressed RANKL‐induced osteoclast‐related protein expression

Osteoclast differentiation and function cannot be separated from the expression of a lot of related symbol protein, such as Cathepsin K, CTR, MMP‐9, TRAP and NFATc1. So, we determined the infect of Cytisine on RANKL‐induced NFATc1 and other related protein by Western blot. As our expected, results indicated that RANKL increased above protein expression at day 1 or day3 and Cytisine prominently diminished CTR and TRAP at day 1 while suppressed Cathepsin K, MMP‐9 at day 3. Interestingly, the Cytisine suppressed protein expression of NFATc1 from day 1 to day 5, especially at day 1. (Figure 4B,C).

### 3.7 Cytisine inhibited RANKL‐induced activation of MAPK signalling pathway

**FIGURE 5:**
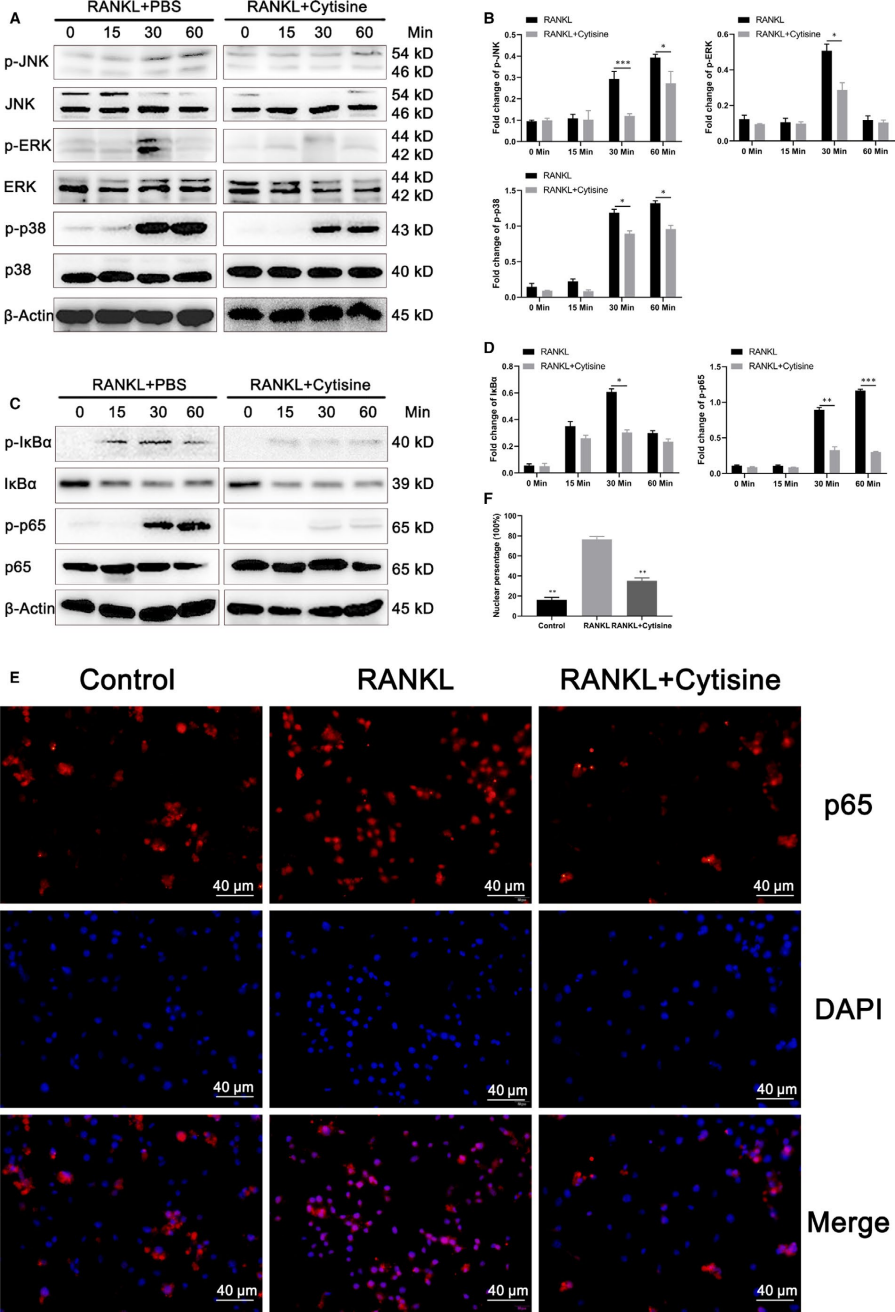
Cytisine inhibited RANKL‐induced activation of MAPK and NF‐κB signalling pathway. A, Representative images of Western blot demonstrating the effect on phosphorylation of JNK, ERK, p38 and total protein induced by RANKL with or without Cytisine at different time point. B, Quantification of osteoclastogenesis‐related protein and JNK/EKR/p38 MAPK pathway protein markers expression levels. C, Representative images of Western blot demonstrating the effect on phosphorylation of IκBα, p65 and total protein induced by RANKL with or without Cytisine at different time point. D, Quantification of osteoclastogenesis‐related protein and IκBα, p65/NF‐κB pathway protein markers expression levels. E, The immunofluorescence staining was performed of location for p65 in the presence or absence of RANKL in RAW 264.7 cells. F, The ratio of fluorescence intensities at nuclear site relative of whole‐cell fluorescence intensity. Data are presented with mean ± SD, *P < 0.05, **P < 0.01

It is important that RANKL‐induced MAPK activation for osteoclastogenesis and function. To detect whether Cytisine suppresses MAPK‐mediated osteoclastogenesis following the stimulation of RANKL on RAW264.7 cells, the JNK, ERK and p38 phosphorylation protein were examined by Western blot analysis. The results demonstrated that Cytisine suppressed the RANKL‐induced phosphorylation of JNK, ERK and p38 in 30 to 60 minutes (Figure 5A,B), especially phosphorylation of ERK in 30 minutes had a significant suppression. These results implied that Cytisine may inhibit RANKL‐induced activation of JNK‐ERK‐p38 which belong to MAPK signalling pathway in osteoclasts.

### 3.8 Cytisine suppressed RANKL‐induced activation of NF‐κB signalling pathway

Besides of the MAPK signalling pathway, NF‐κB signalling pathway may take part in the modulation of osteoclast proliferation and function. To determine whether Cytisine suppresses NF‐κB‐mediated osteoclastogenesis, two different ways were performed for NF‐κB activation. Firstly, we have been suggested that Cytisine could suppress RANKL‐induced phosphorylation and degradation of NF‐κB/IκBα and NF‐κB/p65. The results of Western blot assays were shown in Figure 5C,D that with the RANKL stimulation, phosphorylation of IκBα and p65 were activated at 15 or 30 minutes, respectively; then, both of them were inhibited almost at the same time point after added Cytisine. Secondly, the immunofluorescence staining was performed of location for p65 in the presence or absence of RANKL on RAW 264.7 cells. As shown in the Figure 5E, most p65 were inactive, unphosphorylated and located in the cytoplasm without of RANKL. Whereas, with the presence of RANKL, almost all p65 was transferred into nucleus after 30 minutes of stimulation while this phenomenon was blocked when Cytisine and RANKL were incubated together. However, we examined the ratio of fluorescence intensities which indicated the fluorescence intensity at nuclear site relative of whole‐cell fluorescence intensity (Figure 5F). Therefore, the results implied that Cytisine could suppressed RANKL‐induced activation of NF‐κB signalling pathway.

### 3.9 Cytisine suppressed RANKL‐induced activation of PI3K‐AKT‐NFATc1 signalling pathway

**FIGURE 6:**
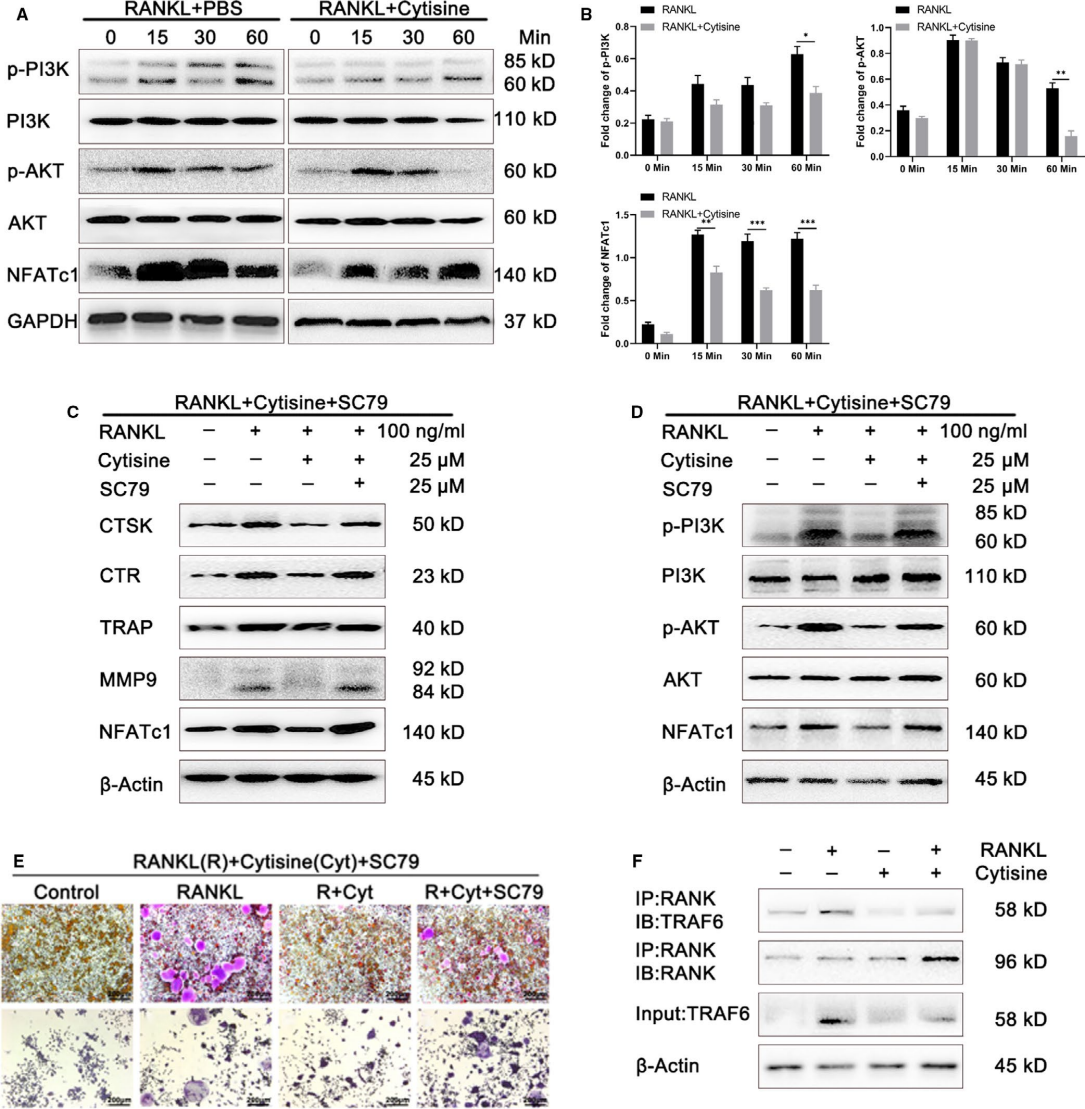
Cytisine suppressed RANKL‐induced activation of PI3K/AKT/NFATc1 signalling pathway and for which rescued by SC79, furthermore, Cytisine suppressed RANKL‐induced RANK‐TRAF6 association. A, Representative images of Western blot demonstrating the effect on phosphorylation of PI3K, AKT and total NFATc1 protein induced by RANKL with or without Cytisine at different time point. B, Quantification of osteoclastogenesis‐related protein and PI3K/AKT/NFATc1 pathway protein markers expression levels. C, Rescue assay was performed to detect the osteoclastogenesis‐related protein levels by activation SC79. D, Rescue assay was performed to illustrate the phosphorylation of PI3K, AKT and total NFATc1 protein levels by activation SC79. E, Rescue assay was performed onto BMMs cells and RWA264.7 cells to measure whether osteoclastogensis was reversed by activation SC79. F, Co‐Immunoprecipitation was performed to measure Cytisine that suppressed the association of RANK and TRAF6. Data are presented with mean ± SD, *P < 0.05, **P < 0.01, ***P < 0.001

PI3K and AKT signalling pathway act as a downstream objective which play a vital role in RANKL‐induced osteoclastogenesis differentiation and function. To determine whether Cytisine suppressed osteoclastogenesis via PI3K and AKT signalling pathway after incubated with RANKL on RAW264.7 cells, we assessed the phosphorylation of PI3K, AKT and total NFATc1 via Western blot analysis. As shown in the Figure 6A,B, the phosphorylated PI3K, phosphorylated AKT and total NFATc1 which belong to PI3K‐AKT‐NFATc1 pathway illustrated a significant increase on RANKL stimulation. Whereas, this phosphorylation was partly suppressed with the treatment of Cytisine. Therefore, these results implied that Cytisine can suppress RANKL‐induced activation of PI3K‐AKT‐NFATc1 signalling pathway.

### 3.10 Rescue assay of osteoclastogenesis‐related protein and PI3K‐AKT‐NFATc1 pathway by activator SC79

Through above work, we found that Cytisine can suppress RANKL‐induced osteoclast‐related protein and activation of PI3K‐AKT‐NFATc1 signalling pathway. Whether is there something could reverse this inhibited situation? So, we select the AKT activator SC79 to detect if it has the rescue effect for osteoclastogenesis‐related protein and PI3K‐AKT‐NFATc1 pathway. As we expected, firstly, we found that osteoclast‐related proteins were up‐regulated after added the SC79 into Cytisine group reduced by RANKL through Western blot (Figure 6C). Secondly, we also observed that the activation of p‐PI3K, p‐AKT and NFATc1 were all up‐regulated after interfered with SC79 and RANKL (Figure 6D). Lastly, the rescued phenomenon for BMMs cells (Figure 6E‐a,b,c,d) and RAW264.7 cells (Figure 6E‐e,f,g,h) which induced by RANKL combination of Cytisine with or without SC79 (Figure 6E) were similar to former results. Thus, above results illustrated that the activator SC79 combination with RANKL can rescue the negative situation of Cytisine for RANKL‐induced osteoclast‐related protein expression and PI3K‐AKT‐NFATc1 pathway.

### 3.11 Cytisine suppressed RANKL‐induced RANK‐TRAF6 association

From the above results, the MAPK, NF‐κB and PI3K‐AKT pathways showed us the similar regulation of molecule mechanism. It was known that the TRAF6 which was recruited by RANK while activated by RANKL, we then wanted to detect whether Cytisine affected the relationship of RANK and TRAF6 through coimmunoprecipitation assay. RAW 264.7 cells were planted into the 6 cm dish at presence or absence of Cytisine and then added with or without RANKL. The cells lysates were harvested for immunoprecipitation with anti‐RANK and then blotted with anti‐TRAF6 (Figure 6F). The results illustrated that RANKL could promote the relation between RANK and TRAF6; however, Cytisine can inhibit this action. Interestingly, Cytisine can also suppress the action of RANK and TRAF6 at absence of RANKL, implying that Cytisine maybe has an effect for RANKL‐induced recruitment of TRAF6 by RANK at endogenous cells.

### 3.12 Cytisine prevented bone loss by suppressing osteoclast activity in vivo

**FIGURE 7:**
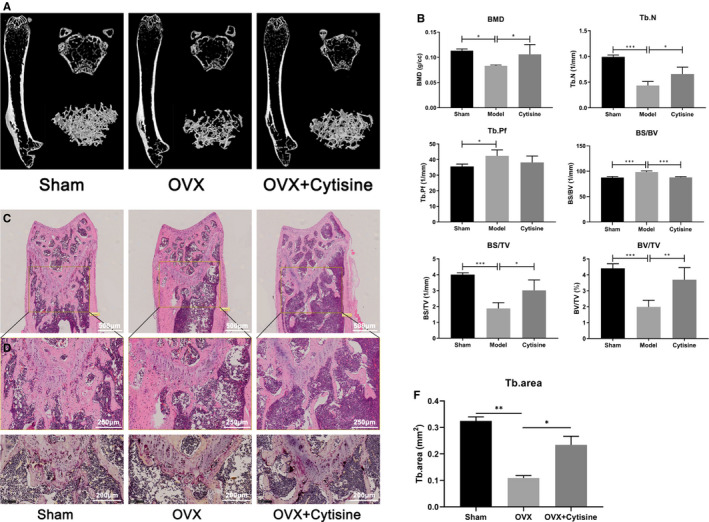
Cytisine prevented bone loss by suppressing osteoclast activity in vivo. A, Representative image of 3D reconstruction μ‐CT of the mouse femur bone μ‐architecture in each group. B, Bone mineral density (BMD), Trabecular number (Tb.N), Trabecular pattern factor (Tb.Pf), Bone surface per volume ratio (BS/BV), Bone surface density (BS/TV), Percent bone volume (BV/TV) were analysed by μ‐CT Skycan CTAn software. (C, D&E) Representative H&E (C&D) and TRAP (E) staining of distal femurs in each group. (F) Quantification of trabecular area per selected field. Scale bar were 500 μm in Figure 7C, 250 μm in Figure 7D and 200 μm in Figure 7E. Data are presented with mean ± SD. *P < 0.05, **P < 0.01

According to above study of Cytisine inhibited RANKL‐induced osteoclast in vitro, it is necessary to explore the potency in vivo that using OVX mice model to mimic post‐menopause osteoporosis in women. Micro‐computed tomography (μCT) scanning and 2/3‐diamention (2D/3D) reconstruction were performed to examine evident on bone loss. The results indicated that at 8 weeks post‐operation, compared with sham‐treated group, OVX model group mice displayed significant trabecular bone loss while there was contrary condition of OVX + Cytisine‐treated group mice that bone loss was markedly prevented, as shown by Micro‐CT (Figure 7A), the 2D/3D recon‐structure were measured through trabecular BMD, Tb.N, Tb.Pf, BS/BV, BS/TV and BV/TV which shown in Figure 7B.

We further explored whether Cytisine suppressed OVX bone loss via inhibited osteoclast differentiation. Compared with OVX model mice, the H&E staining for trabecular density (Figure 7C,D,F) and TRAP‐positive multinucleate cells in distal femurs were significant ameliorated of OVX + Cytisine‐treated mice (Figure 7E). According to data, that Cytisine prevented bone loss in OVX mice by suppressing osteoclast activity more than by encouraging osteoblast in vivo.

## 4 DISCUSSION

**FIGURE 8:**
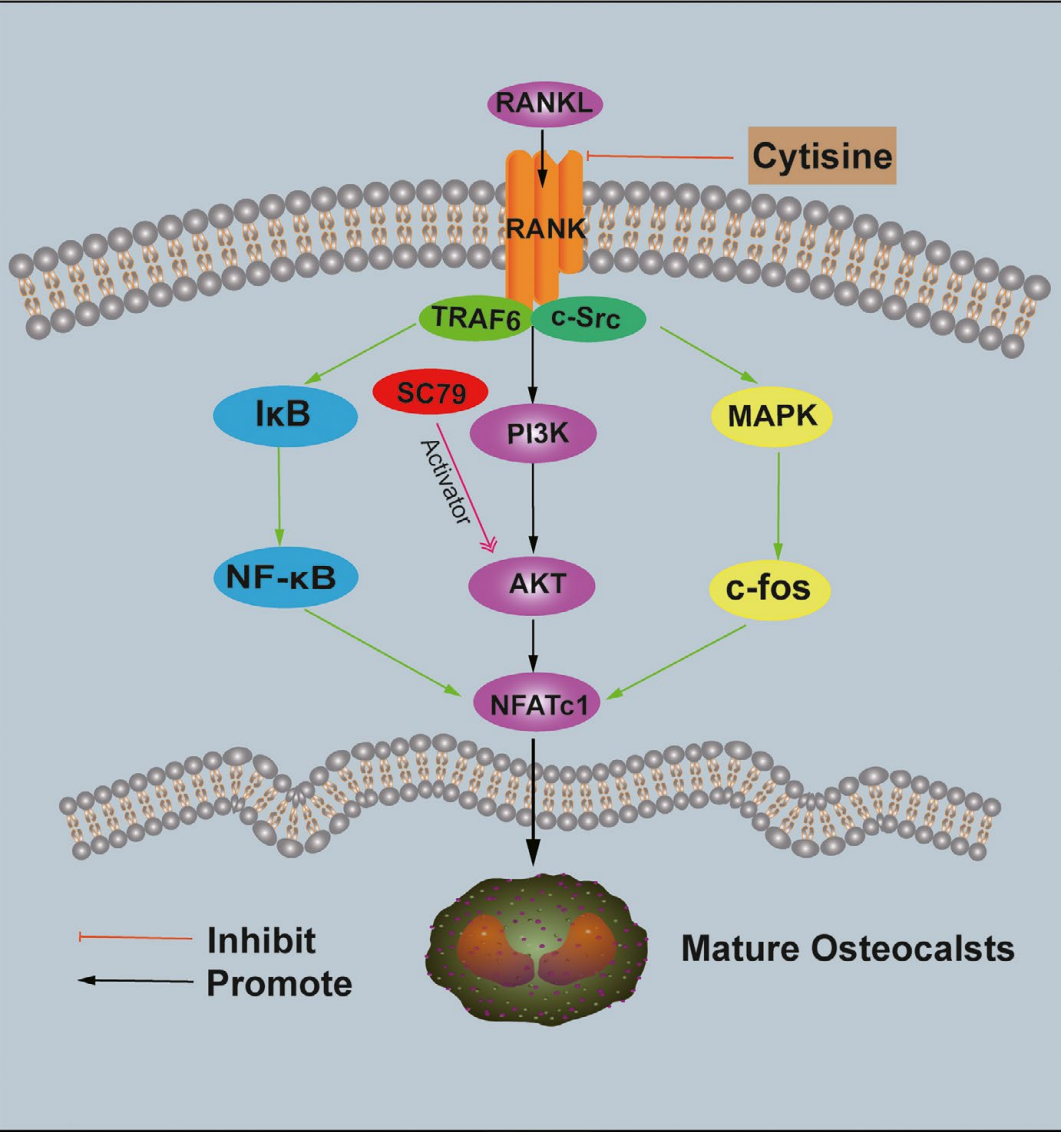
A schematic diagram of the mechanism of Cytisine suppressed osteoclasts differentiation, proliferation and function

In our present study, we illustrated that Cytisine could suppress bone loss by diminishing the activity of osteoclastogenesis in vivo and vitro. Furthermore, we introduced the effect of Cytisine on oestrogen deficiency osteoporosis and bone metabolism which via ovariectomy mice model to mimic the whole process. These downstream typical pathways like as MAPK, NF‐κB and PI3K‐AKT were activated by RANKL during osteoclast precursor cells differentiation and function which were inhibited by Cytisine completely (Figure 8). Because of undoubted effect of Cytisine on protecting bone mineral, according to its structure, it is necessary to detect whether the Cytisine has better anti‐osteoporotic activities.

Oestrogen deficiency is the general phenomenon in older women which could cause bone mineral loss, pathological fracture and increased formation and activation of osteoclast.^21^ Continuous and gradually bone resorption of osteoclast could lead to metabolic bone disorder, such as PMOP.^22^ Thus, suppressing osteoclast differentiation and activation may be a potential strategy for PMOP treatment.^23^ Therefore, to inhabit oestrogen withdrawing and osteoclast activation may provide us a choice for PMOP treatment and restoration.

According to amount of previous reports, numerous biological plant active monomers root in nature products has been studied to display inhibitory effects on osteoclast activation and differentiation.^24^, ^25^, ^26^, ^27^ Cytisine is an alkaloid that was found in plants and seeds of the Leguminosae (Fabaceae) family and has been shown to have several multipotent pharmacological activities.^13^, ^14^, ^15^, ^16^, ^17^, ^18^, ^19^, ^20^ However, there are deficient messages focusing the effect on osteoclastogenesis. In this study, we illustrated the suppression effect of Cytisine on osteoclast differentiation and activation because of RANKL stimulation. After treatment of Cytisine, OVX‐induced bone loss was obvious diminished for mouse model. Thus, Cytisine will be possible to become an osteoprotective agent.

We managed this study to detect effect of Cytisine on PMOP and molecular mechanisms. In vivo, bone loss was prevented significantly by Cytisine for ovariectomy mice after 8 weeks treatments, as displayed in H&E, TRAP staining and micro‐CT scanning at distal femur. In vitro, RANKL and M‐CSF dual‐induced osteoclast differentiation and F‐actin ring formation were significantly inhibited by Cytisine for BMMs and RAW264.7 cells. Meanwhile, those above two types differentiated osteoclast related marker genes including NFATc1, Cathepsin K, MMP9 and TRAP were also inhibited by Cytisine. Therefore, we conjectured that Cytisine prevented OVX‐induced bone loss may be via suppressing osteoclastogenesis.

RANKL is very important for osteoclastogenesis differentiation and function.^28^, ^29^ When RANKL recognized and engaged RANK on pre‐osteoclast cells, the downstream pathways such as MAPK, NF‐κB and PI3K/AKT were all activated and TRAF6 was recruited. TRAF6 could exert dominant role in signal transduction.^30^, ^31^, ^32^ Upon binding of RANKL and RANK, those above signalling pathways have been studied to modulate osteoclast survival and differentiation that were related to both RANKL and M‐CSF. PI3K/AKT‐NFATc1 axis was vital for RANKL‐induced activation of osteoclasts. From our study, the phosphorylation of JNK, ERK, p38, p65, IκBα and total NFATc1 protein activation was inhibited by Cytisine. The RANKL‐induced PI3K activation and AKT signalling were also inhibited by Cytisine. Meanwhile, if we added AKT activator SC79 into Cytisine group that an interesting phenomenon was occurred, which SC79 could rescue the suppression of Cytisine onto RANKL‐induced osteoclastogenesis‐related protein expression and PI3K‐AKT‐NFATc1 pathway (Figure 8). So, we adventurously conjectured that (a) there are several signalling pathways exert the modulation for the RANKL‐induced osteoclastogenesis differentiation and proliferation at endogeny; (b) there may at least one signalling pathway plays a leading role in function of osteoclast.

NFATc1 is an important transcription factors in osteoclast differentiation.^10^ NFATc1 is an NFAT family member that has been reported to modulate expression of several osteoclast‐related genes including TRAP, cathepsin K and MMP‐9.^33^ In pro‐osteoclast cells, NFATc1 can accelerate transcriptional processes. Therefore, the factor NFATc1 deficiency could cause severely osteopetrotic phenotypes.^34^ These key genes were all up‐regulated by RANKL‐induced osteoclastogenesis and were down‐regulated by Cytisine treatment, indicating that Cytisine affected NFATc1 and downstream gene expression.

Because of osteoblasts are vital for bone formation, structure and mineralization,^35^, ^36^, ^37^, ^38^ the effect on osteoblast of Cytisine was managed at our study. However, there were no significant statistics between Cytisine treatment group and control group below 25 μmol/L dosage. In a word, followed our data, we speculated that Cytisine has no significant effects on differentiation and mineralization of osteoblast at the dosage rang of 0‐25 µmol/L.

Limitation of our study that may be the guideline for future work. First, the molecular signalling mechanism has not yet to be elaborated. Cytisine could suppress MAPK, NF‐κB and PI3K‐AKT signalling pathways, implied that Cytisine maybe through several pathways exert function of anti‐osteoporosis. Second, the cellular interaction target has not been explained and need to be explored at next work. Third, Cytisine displayed evident inhibitory effect on osteoclast, it possibly has therapeutic effect on osteoclast‐related diseases, such as osteoarthritis, rheumatoid arthritis and ossifying myositis which needs to be clarified in the further. Finally, in vivo proved that Cytisine could obviously prevent OVX‐induced bone loss and suppress osteoclast activation. However, it brings the question of what effects Cytisine has on osteogenesis and bone formation.

Owning to without saving the serum of mice, so there are deficiency data of serum levels for OCN, OPG, Runx2 and osteogenesis related gene markers, so we have responsibility to explore the relationship of Cytisine and osteogenesis in the future. However, followed the whole study data, we speculated that the level of OPG may be down‐regulated in the OVX model group, while be up‐regulated in the OVX + Cytisine treatment group.

To sum up, our study illustrated that Cytisine could act as an effective and novel agent for PMOP by inhabiting partly cellular signalling pathway. This may supply a reference to exploit novel therapeutic effect drugs for osteoclast‐associated disorders.

## CONFLICT OF INTEREST

The authors declare no conflicts of interest.

## AUTHOR CONTRIBUTION

**Zhi Qian:** Conceptualization (equal); Data curation (equal); Formal analysis (equal); Methodology (equal); Software (equal); Validation (equal); Visualization (equal); Writing‐original draft (equal); Writing‐review & editing (equal). **Zeyuan Zhong:** Data curation (equal); Formal analysis (equal); Methodology (equal); Software (equal); Visualization (equal). **Shuo Ni:** Data curation (equal); Methodology (equal); Validation (equal). **Dejian Li:** Conceptualization (equal); Project administration (equal); Writing‐review & editing (equal). **Fangxue Zhang:** Data curation (equal); Methodology (equal); Validation (equal). **Ying Zhou:** Data curation (equal); Validation (equal). **Zhangrong Kang:** Data curation (equal); Validation (equal). **Jun Qian:** Conceptualization (equal); Funding acquisition (equal); Supervision (equal). **Baoqing Yu:** Conceptualization (equal); Funding acquisition (equal); Investigation (equal); Project administration (equal); Supervision (equal); Validation (equal); Writing‐review & editing (equal).

## Supporting information

Fig S1

## Data Availability

All data, models and code generated or used during the study appear in the submitted article.
