# Supplementary figures and images for: Cytisine attenuates bone loss of ovariectomy mouse by preventing RANKL‐induced osteoclastogenesis
Source: J Cell Mol Med. 2020 Aug 13;24(17):10112–27. doi: 10.1111/jcmm.15622 (PMC7520284; doi:10.1111/jcmm.15622)

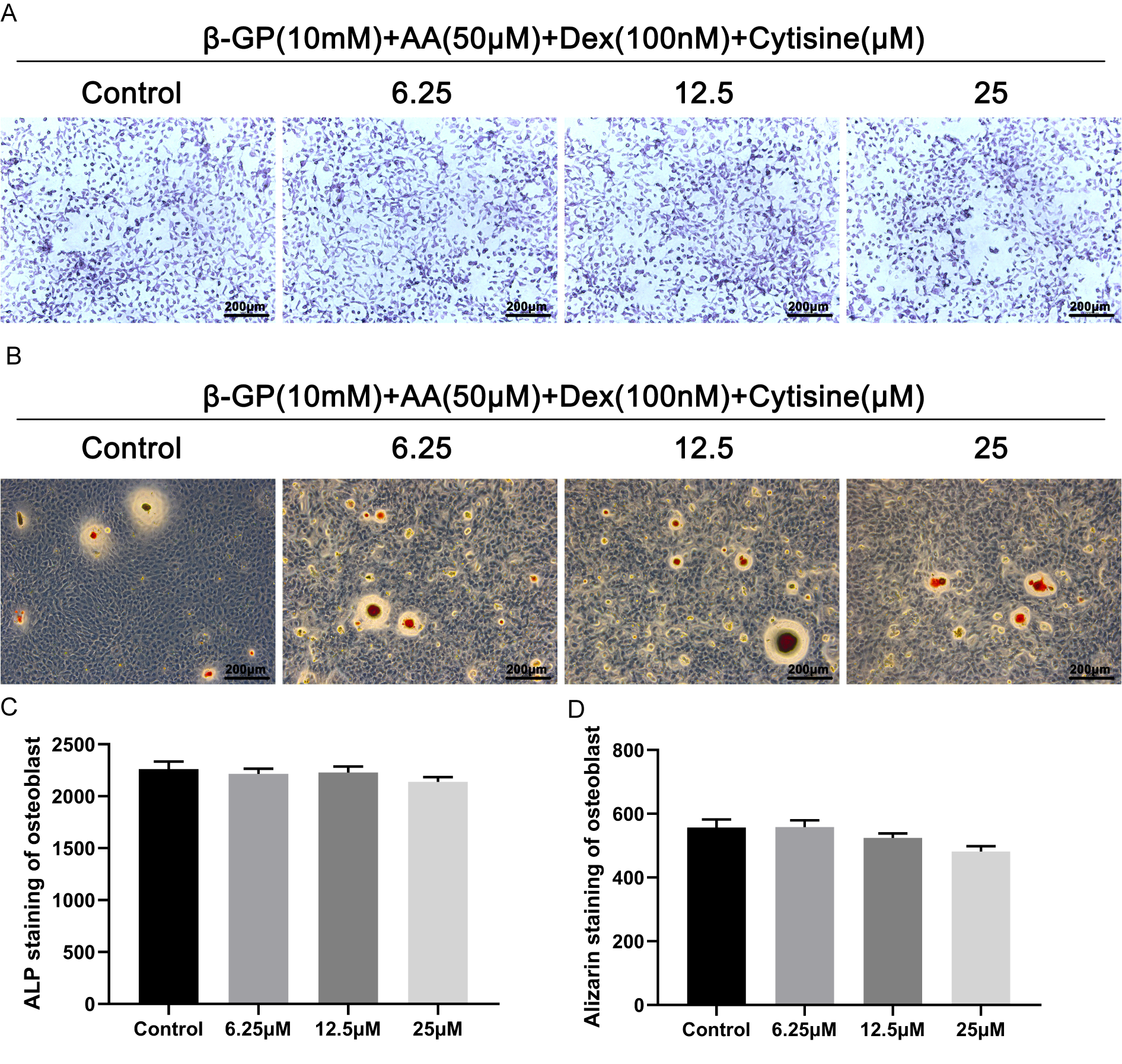

Supplement: Supplementary file 1 — Fig S1 [file JCMM-24-10112-s001.tif]
